# Multiple Extracranial Metastases from Primary Gliosarcoma in a Patient with Two Previous Different Primary Cancers

**DOI:** 10.1155/2019/7849616

**Published:** 2019-09-02

**Authors:** T. Capion, J. Hauerberg, H. Broholm, A. Muhic

**Affiliations:** ^1^Department of Neurosurgery, The Neuroscience Centre, Copenhagen University Hospital Rigshospitalet, Copenhagen, Denmark; ^2^Department of Pathology, Center of Diagnostic Investigation, Copenhagen University Hospital Rigshospitalet, Copenhagen, Denmark; ^3^Department of Oncology, The Finsen Centre, Copenhagen University Hospital Rigshospitalet, Copenhagen, Denmark

## Abstract

Gliosarcoma (GS) constitutes a minor fraction of primary glioblastoma (GBM), which is the most frequent malignant brain tumor in adults. Despite the fact that malignant gliomas are highly invasive, extracranial metastases are very rarely seen, and the mechanisms behind extracranial dissemination are still unclarified. We report a case of a 55-year-old male with a prior history of two distinct primary cancer types who, as a third independent type, developed GS with penetrating tumor growth to the skull and subcutaneous soft tissue via explosive spreading through a titanium net as well as extracranial metastases to the lumbar spine, paravertebral musculature, and most likely the right lung. The case illuminates the clinical challenge of diagnosing extracranial metastases from primary GBM and GS as these are still unexpected, especially in cases with possible competing diagnoses.

## 1. Introduction

In adults, glioblastoma accounts for approximately 15% of all intracranial neoplasms and approximately 45-50% of all primary malignant brain tumors and is thus the most frequent type [1]. It has a very aggressive natural history and an unfavorable prognosis with an estimated mean survival of 10-15 months from time of diagnosis despite multimodal treatment [2, 3].

Gliosarcoma is a rare variant of GBM, representing approximately 2% of all GBM, and consists of a biphasic tissue pattern of glial and mesenchymal cells corresponding histologically to WHO grade IV [1, 4, 5]. Formerly referred to as *glioblastoma with sarcomatous components*, gliosarcoma is considered an independent disease entity in the recent World Health Organization (WHO) classification [1, 6]. It affects males nearly twice as often as females and is usually diagnosed in the fourth to sixth decade of life [7, 8]. Despite its known aggressiveness, extracranial metastases from GBM are seen as rarely as in 0.4-0.5% of cases, with still only around 200 case reports published worldwide describing this phenomenon [9, 10].

## 2. Case Presentation

We present the case of a 55-year-old male with a prior history of a neuroendocrine tumor and colon cancer.

In January of 2014, the patient was diagnosed with an adnexal skin tumor on the cheek, which was macroradically removed and subsequent histological analysis confirmed the diagnosis of a primary neuroendocrine tumor (NET). Two years of follow-up revealed no signs of metastases or relapse.

In June of 2016, the patient was diagnosed with an intestinal adenocarcinoma classified as T3N1V0 (*TNM Classification of Malignant Tumors*), with spreading to 2 out of 28 lymph nodes and no histological signs of metastases from the NET. He received 6 months of adjuvant chemotherapy (FOLFOX regime), with initial supplement of oxaliplatin, which was discontinued after 6 series due to dysesthesia to the extremities. One year of follow-up revealed no signs of metastases or relapse.

**Figure 1:**
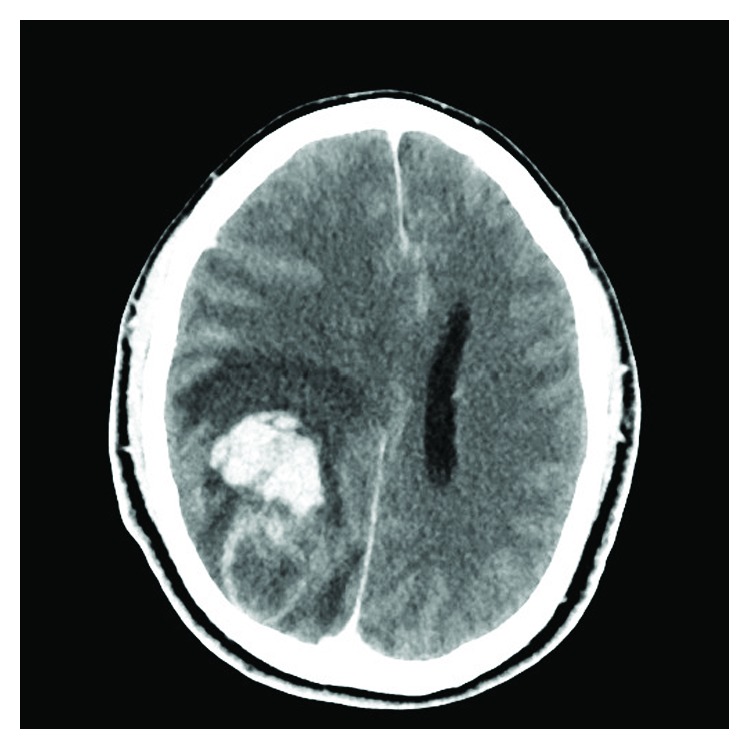
Preoperative post contrast-enhanced axial cut of the CT scan of the brain showing tumor masses located parieto-occipitally in the right hemisphere surrounded by perifocal edema and a 54 × 31 mm intraparenchymatous bleeding with affection of midline structures.

In May of 2018, the patient was admitted due to one week of gait abnormality and one day of intense headache. He was, at time of hospitalization, drowsy with Glasgow coma score 14 (*eyes* 3, *verbal* 5, *motor response* 6) with left-sided homonymous hemianopsia and left-sided hemiparesis. A contrast-enhanced CT scan of the brain revealed a tumor-suspected mass in the right parieto-occipital region surrounded by hemorrhage and edema, which caused mass affection of midline structures, basal cisterns, and sulci of the brain (Figure 1). Acute removal of the hemorrhage and macroradical tumor resection was performed via right-sided parieto-occipital craniotomy.

**Figure 2:**
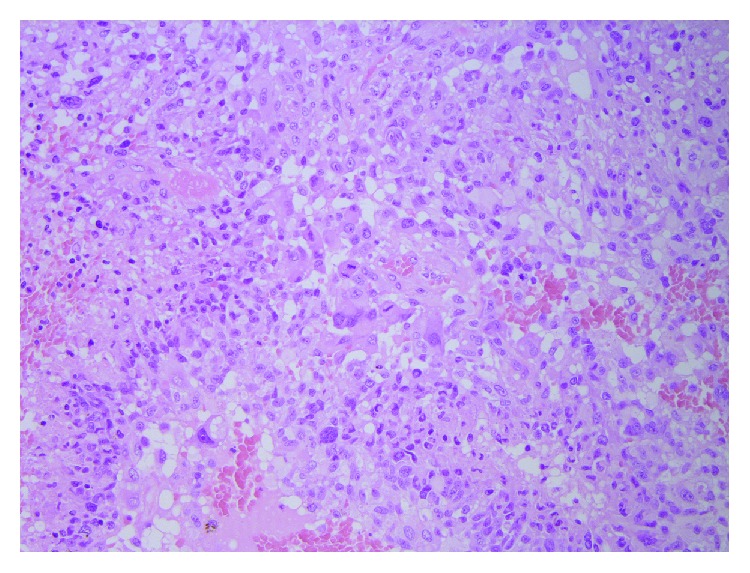
Histology showing a pleomorphic glial tumor, with numerous mitoses, malignant vascular proliferation with vascular thromboses, necrosis, and pseudopalisading tumor cells (HE, ×200).

**Figure 3:**
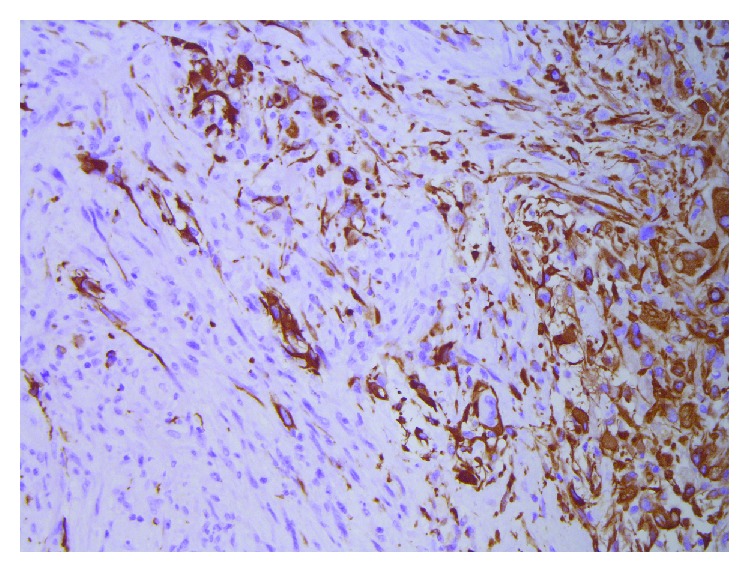
GFA staining (×200) of the same glial tumor showing biphasic tumor tissue with the mesenchymal component being negative and the more obvious glial component being positive in GFA, compatible with gliosarcoma, WHO grade IV.

Histological analysis revealed the tissue to be of malignant glial origin with astrocytic cells with pleomorphic nuclei and numerous mitoses, in a pattern of microvascular proliferation, thrombosed vessels, and guirlande-like necrosis. Immunohistochemical analysis showed extensive positive staining for GFA, olig2, map2, and p53. IDH mutation was negative, ATRX was normal, and Ki-67 was high. These findings were conclusively compatible with the diagnosis of GBM, WHO grade IV, IDH-wild type. PCR sequencing showed a 2% methylation of the O^6^-methylguanine-DNA methyltransferase (MGMT) gene, which with a cutoff value of 10% methylation, was concluded to be negative (Figures 2 and 3).

The patient was according to standard care for the treatment of GBM referred to fractionated radiotherapy (30 fractions of 2 Gy) and concomitant chemotherapy with temozolomide for which he initially responded well. He afterwards received adjuvant temozolomide.

An MRI of the brain was performed 6 months after the second series of adjuvant temozolomide due to symptom progression. The scan revealed progression of tumor masses around the previous resection cavity, and reoperation with fluorescence-guided surgery using 5-aminolevulinic acid (5-ALA) was performed. Macro radical resection was at this point not possible due to comprehensive spreading of tumor masses, which was intraoperatively discovered to have infiltrated the dura mater and skull. The latter prompted removal of a part of the skull and insertion of a titanium net.

**Figure 4:**
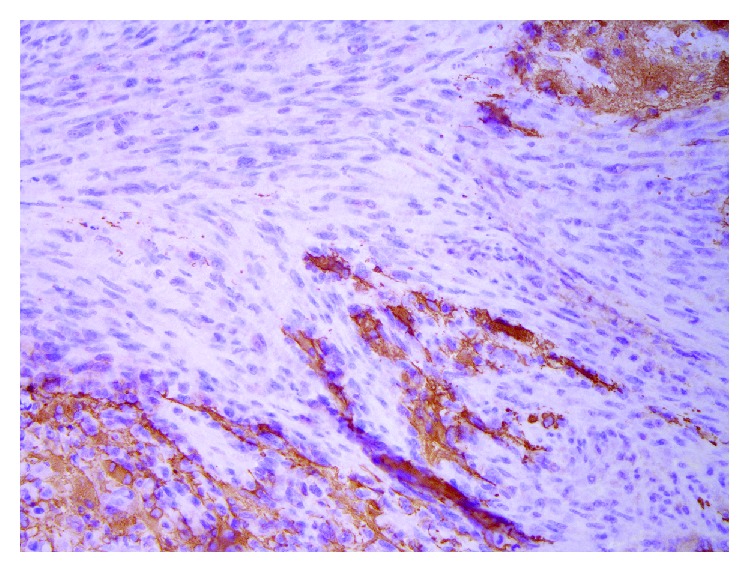
Histology from the reoperation (GFA, ×200) revealing a glial tumor with extensive sarcomatous components and more sparse astrocytic tumor cells being positive in GFA, compatible with regrowth of gliosarcoma, WHO grade IV.

Subsequent histological analysis confirmed the diagnosis of relapse of glioblastoma, yet now with extensive sarcomatous differentiation compatible with gliosarcoma, WHO grade IV, IDH-wild type. Microscopically, dura mater, leptomeninges, and cortical brain parenchyma were seen infiltrated by sarcomatous spindle cells with pleomorphic nuclei, numerous mitoses, and extensive necrosis. Immunohistochemical staining was negative for GFA, Olig2, and map2 and extensively positive for p53. IDH-mutation was negative (Figure 4).

**Figure 5:**
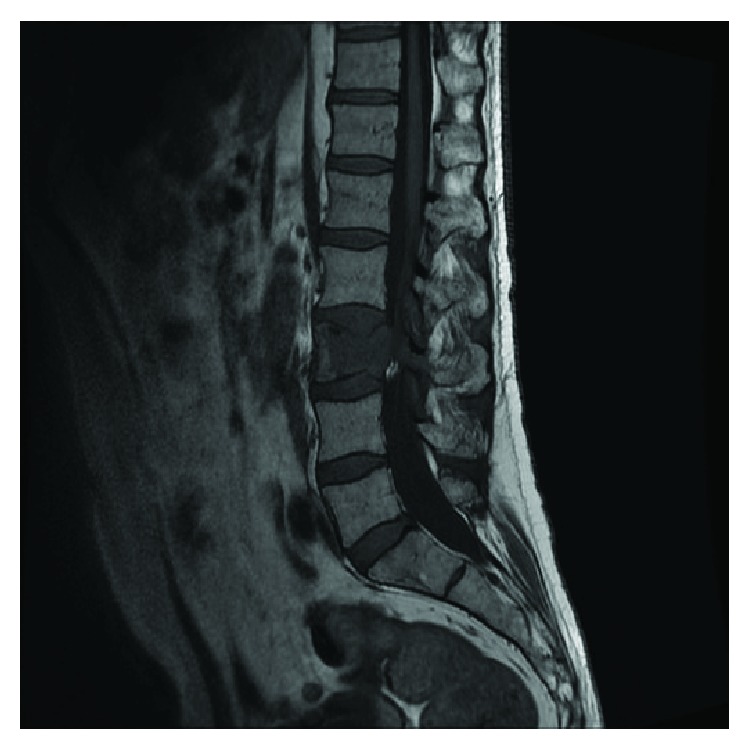
Sagittal T1-weighted MRI of the vertebral column without contrast enhancing showing tumor masses in the 3rd lumbar vertebrae (L3) with partial collapse of the corpus of L3 and tumor growth to the right paravertebral muscle.

**Figure 6:**
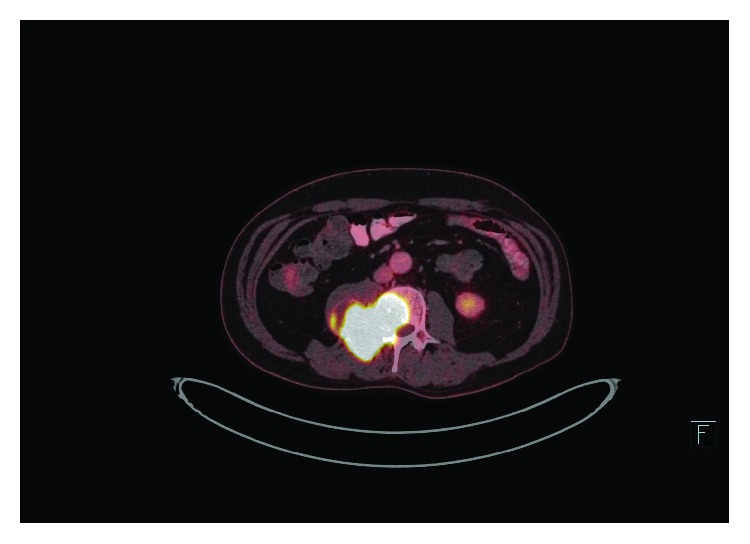
Fused FDG/PET-CT scanning at level of L3/L4 7 months after diagnosis of GBM showing substantial metabolically active metastases in and around L3 and surrounding paravertebral muscle.

**Figure 7:**
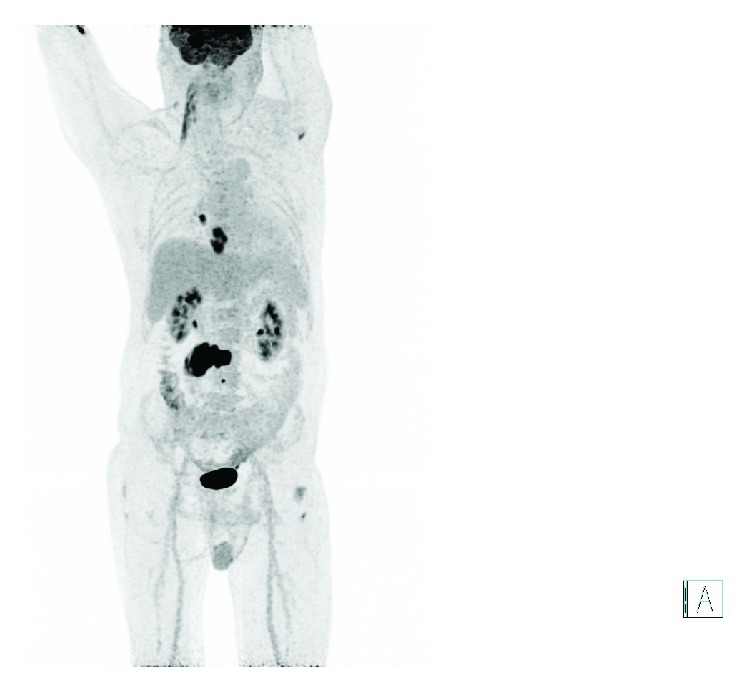
Frontal maximum intensity projection (MIP) image of whole body FDG PET scanning identifying disseminated metastatic spread to mediastinal lymph nodes, right lung, vertebral bone, and right paravertebral muscle. Physiological excretion to the intestines, kidneys, and bladder.

One week after the reoperation, the patient gradually developed radiating lower back pain. An MRI of the lumbar column revealed a mass around the 3rd and 4th lumbar vertebrae (L3/L4) with epidural and muscular ingrowth, suspected radiographically to be of metastatic origin. A whole body FDG/PET-CT scan (*fluorodeoxyglucose/positron emission tomography-computed tomography*) was performed which besides the known mass in the lumbar spine revealed a round infiltrate in the right lung, enlarged lymph nodes around both lungs and in the mediastinum, and embolisms to all three lobes of the right lung (Figures 5–7).

**Figure 8:**
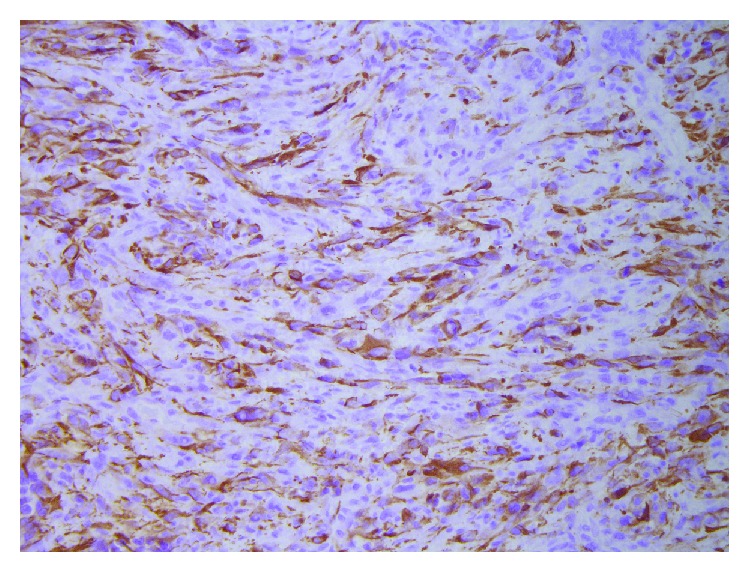
Histology from tumor tissue in the lumbar column confirmed a pleomorphic biphasic glial tumor as diagnosed earlier, compatible with metastases from gliosarcoma, WHO grade IV (GFA, ×200).

Due to the risk of medullary cross-section syndrome, surgical decompression of L3/L4 with instrumental stabilization was performed. Histological examination of biopsies from the tumor mass in L3 showed tissue which constituted of glial and spindle-shaped cells with microvascular proliferation and necrosis. Tumor cells were pleomorphic and polynuclear. Ki-67 was high, ATRX was normal, and IDH-mutation was negative. PCR sequencing showed a 2% methylation of the MGMT gene. Morphologically and immunohistochemically, these findings were compatible with relapse of GS, with no evidence of metastases from the previous NET or colon cancer (Figure 8).

Due to the patient's general condition and the prognosis of the primary illness, it was decided not to biopsy from the mass in the right lung, and he was referred to radiotherapy towards the remaining tumor masses in and around L3. He was at this time in a general deteriorating condition, and therefore solely considered to be a candidate for palliative irradiation without further chemotherapy treatment.

**Figure 9:**
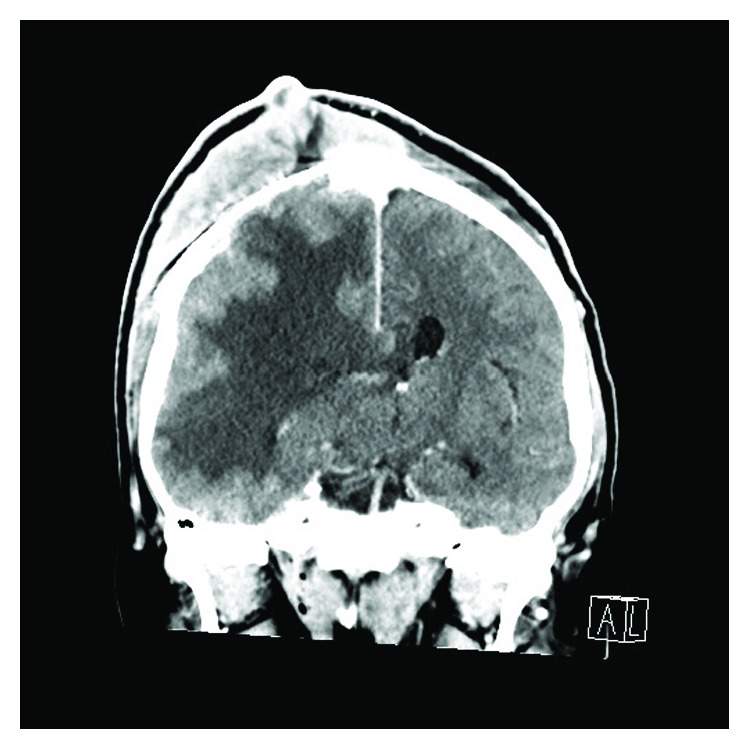
Coronal cut of CT scan of the brain showing tumor masses infiltrating the skull, subcutaneous soft tissue, and skin overlying the previous surgical cavity in which perifocal edema is causing midline shift towards the left hemisphere.

A contrast-enhanced CT scan of the cranium and brain was performed shortly after due to an aggravating headache and formation of pouches under the wound from the second craniotomy. The scan confirmed pervasive expanding tumor growth through the titanium net, penetrating the skull, and reaching the subcutaneous soft tissue (Figure 9).

The patient passed away 8 months after the initial diagnosis of GBM after a process of rapidly progressed illness which ultimately resulted in penetrating tumor spread to the skull and subcutaneous soft tissue due to explosive growth of the primary tumor, as well as multiple extracranial metastases to the lumbar spine, paravertebral musculature, and most likely the right lung and lymph nodes in the mediastinum.

## 3. Discussion

Although described for the first time in 1928, the mechanisms behind the infrequent development of extracranial metastases from primary GBM remain unclear [10]. It has been suggested that malignant cells rarely escape the cerebral environment on the basis of several factors, including the lack of a cerebral lymphatic system, the protection of cerebral venous vessels by dura mater, the blood-brain barrier, and the overall short survival time for patients with GBM [9].

Hematogenous dissemination is one of the most sought-after explanations for the enabling of this cell escape [5, 10–12]. Lung metastases have been estimated to occur in around 60% of those affected by extracranial metastases from primary GBM, in lymph nodes in just above half (50%), whereas bone metastases are seen in approximately 30%, of which metastases to the vertebrae constitute the majority (70%) [12]. This diverse settlement of malignant cells in various anatomic locations and types of tissues confirms a versatile route for the intracerebral malignant cells to migrate. This route could be hematogenous or lymphatic or possibly via circulation of cerebrospinal fluid to structures in immediate relation to dura mater and its vessels, in this case, namely, the lumbar vertebrae.

Müller et al. found in a study from 2014 that more than 20% of patients with primary GBM had circulating tumor cells in the peripheral blood, confirming the potential of these cells to escape the intracerebral environment and survive for a not negligible amount of time in the peripheral bloodstream [5, 12]. Müller et al. did, however, not identify a significant correlation between surgical manipulation of intracerebral tumor tissue and the presence of circulating tumor cells in the peripheral blood, downplaying the importance of this factor, which has on multiple previous occasions been suggested as a probable way for tumor cells to migrate from the intracranial space [5]. The patient in the present case developed extracranial metastases approximately 7 months after primary surgery. At time of reoperation, tumor masses had infiltrated the dura mater, skull, and settled to form metastases in distant internal organs as well as in bone, suggesting an either early escape from the intracranial space, a highly aggressive invasion of foreign tissues, or a combination of the two.

More sparsely described is the relationship between primary GBM and GS and other previous or simultaneously occurring primary malignant tumors and their internal influence on one another.

The three primary cancers diagnosed in the present case were of different tissue origin and not situated in immediate anatomical proximity. Nguyen et al. found in a population-based analysis from 2018 significantly shorter survival in patients with primary GBM who had previously been diagnosed with one or more of a number of different types of primary malignancies, including in the digestive system [13]. This correlation and influence between GBM and GS and other types of primary cancer has been described on case report level previously [14, 15]. An underlying physiological phenomenon linking the development of these parallel malignancies has, to the best of our knowledge, not been identified. Molecular analysis of these cases with, i.e., 850K methylation and NGS, may give some information of whether a genetic, or in other ways physiological, connection between the three individual malignant diseases in the present case exist or not, and if so, of which kind.

The present case is interesting and unique in the setting that GS was the third primary malignant disease diagnosed within a period of 4 years. It originated as a relapse from primary GBM and metastasized in an aggressive disease pattern to numerous extracranial locations. This case illustrates how extracranial metastases from primary GBM, and GS, still constitute a significant diagnostic and therapeutic challenge although the phenomenon is known and described in the literature, especially in a case like the present, in which previous primary malignant diseases challenged the diagnostic picture.
